# Extrusion Effect or Promotion Effect? The Effect of Environmental Regulation on Enterprise Green Innovation

**DOI:** 10.3390/ijerph20031748

**Published:** 2023-01-18

**Authors:** Wei Wang, Hailin Chen

**Affiliations:** 1 Academy of Science and Technology Development, Journal Center, Huazhong Agricultural University, Wuhan 430000, China; 2 School of Economics and Trade, Guangdong University of Foreign Studies, Guangzhou 510006, China

**Keywords:** environmental regulation, green innovation, DID, China

## Abstract

This paper took the policy of China’ Air Pollution Prevention and Control Action Plan as an exogenous shock to reflect the change in environmental regulation intensity. By matching environmental policies with micro data of listed companies in China, this paper explored the effect and mechanisms of environmental regulation on enterprise green innovation. Through constructing difference-in-difference (DID) and difference-in-difference-in-difference (DDD) models, we found the following to be the case: (1) Environmental regulation had a significant positive effect with the green innovation level of Chinese listed companies. (2) Compared with non-regulated industries, this policy has led to a significant increase (5.4%) in the amount of firms’ green patent applications in regulated industries, and the promoting effect was more obvious in key areas that are strictly controlled by this policy. (3) Compared with non-state-owned enterprises, it had a stronger impact on the green innovation of state-owned enterprises. (4) Mechanistic analysis showed that China’s environmental regulation can play a resource compensation effect by increasing environmental protection subsidies for enterprises’ green innovation behaviors. Additionally, it can force firms to increase investment in environmental pollution governance by raising pollution penalties, thus exerting the forcing effect. This paper provides new evidence for Porter’s hypothesis and can provide a reference for developing countries promoting green innovation through environmental policies and regulations.

## 1. Introduction

With the acceleration of industrialization, global warming and environmental pollution have become a global public problem [1]. In 2016, 178 countries signed the Paris Agreement, in which countries commit to take stronger measures to address climate change and environmental pollution. The 2018 World Environmental Pollution Index jointly released by Yale University and other institutions shows that among 180 countries, China’s environmental governance performance ranks 120, and China’s air quality ranks fourth from the bottom [2]. This ranking reflects the severity of environmental pollution in China and highlights the urgency of implementing environmental protection policies. The Chinese government has actively participated in international cooperation related to environmental governance, such as announcing that it would achieve carbon peak in 2035 and carbon neutralization in 2060. To archive this ambition, many effective policies have been implemented by Chinese government to reinforce environmental regulation, such as the Air Pollution Prevention and Control Action Plan promulgate in 2013, the New Environmental Protection Law enacted in 2014, and the central supervision system on ecological and environmental protection established in 2015. Although these policies have obvious differences in the scope of application, implementation difficulty, and effects, all of them have strengthened China’s environmental regulation. Existing studies have shown that a series of environmental regulation policies implemented in China has had a positive effect on reducing environmental pollution [3,4].

Nowadays, most countries have paid increasingly more attention to environmental protection and have implemented stricter policies to prevent pollution. Reducing pollution emission by green innovation has been an inevitable choice for enterprises to achieve sustainable development [5]. Therefore, the relationship between environmental regulation and green innovation has also become a research hotspot in recent years. For example, the Porter hypothesis proffers that governments can internalize the positive externalities of green innovation, thus providing a remedy for the cost of green innovation [6]. That is, environmental regulation can not only protect the environment but can also increase economic benefits of firms through promoting enterprise green innovation [7]. Many empirical research results are consistent with this view. Reiner [8] analyzed the energy-intensive industries in Europe and found that environmental regulations increased energy prices, forcing energy enterprises to reduce energy consumption through innovation, thus promoting green innovation of energy-intensive enterprises. Similarly, Ravetti et al. [9] conducted a study on the energy-intensive container shipping industry and found that environmental regulation policies urge shipping enterprises to reduce energy consumption by developing new products and technologies.

Many empirical studies based on Chinese data are consistent with this view. Using the data of firms in China’s four national eco-industrial parks, Peng et al. [4] revealed that there is a significant positive relationship between environmental rules and firms’ green innovation; in addition, compared with the market-driven rules, command–control environmental rules have a stronger promotion effect. Similarly, Zhong and Peng [10] found that after the implementation of the New Environmental Protection Law in 2014, the green innovation level of Chinese A-Share listed companies have been significantly improved. Taking the Chinese Central Ecological and Environmental Protection Supervision Action as an exogenous practice, Song et al. [11] confirmed the compensation effect and cost effect of environmental regulation on firms’ green innovation, finding that the former effect is stronger than the latter. Moreover, it also showed that, in heavy pollution industries, green invention patents can receive a stronger positive effect from this policy than other types of green innovation. However, some studies found that environmental regulation can also produce a restrictive or crowding-out effect, thus hindering firms’ green innovation. According to this view, environmental regulation will force enterprises to increase investment in pollution control, which will increase the costs of institutional compliance, and the increased costs will crowd out R&D investment [12,13]. This view is also supported by empirical results. Through analyzing U.S. industrial enterprises, Yu et al. [14] found that the more environmental policies are issued, the lower the motivation for firms’ innovation. Kneller and Manderson [15] analyzed the manufacturing industry in Britain and promulgated that in order to comply with the environmental protection rules, firms have to increase the expenditure to decrease pollution emission, which will increase firms’ financial pressure and reduce R&D investment. In addition, using data from the European automotive industry, Barbieri [16] promulgated that environmental regulation has not effectively promoted the progress of pollution emission technology in the automotive industry.

The nonlinear relationship between these two issues has also been revealed by some studies. Using panel data of 235 cities in China, Fan et al. [17] revealed that there is a U-shaped relationship between environmental regulation and regional green innovation. Song et al. [18] obtained the same conclusion through conducting an empirical study with data of China’s provinces. Conversely, Zhao and Sun [19] found that there is an inverted U-shaped relationship between these two variables in eastern China. Namely, slight environmental regulation can effectively stimulate green innovation; however, it will produce an impeding effect when the regulation intensity exceeds the critical value. Therefore, the impacts of environmental regulation on enterprise green innovation remain ambiguous. Even the most popular theory for this issue, the Porter hypothesis, can also be divided into many categories, namely, the narrow hypothesis, weak hypothesis, and strong hypothesis [4]. Yet, many empirical studies did not consider the scope of application, implementation difficulty, and effective time of environmental protection policies when taking them as quasi-natural experiments to measure the changes in environmental regulation. In addition, many studies only take enterprises in some regions or industries as research samples, which restricts the representativeness of the research results [20]. What is more, we usually ignore the low innovation capacity of firms when discussing the situation of developing countries, which may lead to a different performance when faced with government regulation [21]. 

To reveal this relationship clearly, this paper regards China’s Air Pollution Prevention and Control Action Plan as a quasi-natural experiment of environmental regulation and explores its effect on the green innovation level of A-Share listed companies in China. The marginal contributions of this paper are as follows: (1) We constructed a DID model to identify the effect of environmental regulation policies that can alleviate the errors caused by endogenous problems. Although the focal policy is national, it also identifies key areas and industries, which also allowed us to build a DDD model for further research. (2) We also revealed the different effects of environmental regulation on the green innovation level of firms with different property rights. The result proclaims that the promotion effect of this policy is stronger in state-owned enterprises, which is different from the traditional belief that private firms are more innovative. (3) This paper also examined the function mechanisms from the perspective of the resource compensation effect and forcing effect; specifically, it can produce the compensation effect through providing more fiscal subsidies and produce the forcing effect through enforcing firms to improve investment in environmental protection in order to control pollution emissions. This provides a reference for developing countries to develop environmental regulation policies to promote green innovation.

## 2. Policy Background and Theoretical Analysis

### 2.1. Policy Background

Around 2012, China experienced the most serious haze in history, which had a great adverse effect on residents’ work and life [22]. To respond to public pressure and improve atmosphere quality quickly, the Chinese government issued the Air Pollution Prevention and Control Action Plan (referred to as Action Plan hereafter) in September 2013. Compared with other environmental regulation policies, this policy had clear objectives, had specific and feasible measures, and designated the administrative department in charge [23]. After the release of the policy, it was effectively implemented by local governments and made achievements in reducing air pollution emissions. Therefore, the Action Plan is regarded as one of the most successful environmental regulation policies in China [24].

This Action Plan sets clear goals for atmosphere improvement. According to the Action Plan, compared with 2012, the concentration of inhalable particles in cities should be slashed at least by 10% in 2017. Moreover, higher targets have been proposed for some key areas, namely, the Beijing–Tianjin–Hebei, which region should realize a reduction goal of at least 25%; the Yangtze River Delta, which should realize a reduction goal of at least 20%; and the Pearl River Delta, which should realize a reduction goal of at least 15% [23]. Moreover, the annual average concentration of fine particles in Beijing was controlled at about 60 micrograms per cubic meter, and this value was 89.5 micrograms per cubic meter in 2013. The action plan also proposes ten specific policy measures, including both command-controlled type and market-driven type measures [24]. The command-controlled policies include increasing the comprehensive governance of air pollution in industrial enterprises, strictly controlling the new production capacity of industries with high pollution emissions and high energy consumption, accelerating the elimination of under-developed production capacity and controlling the total amount of coal consumption [25]. The market-driven environmental regulation policies include giving play to the regulatory role of the market mechanism, improving the price and tax policies related to environmental governance, and broadening the financing channels for the environmental protection of enterprises [26]. To monitor fulfillment of these policies and stimulate the enthusiasm of the local government in environmental protection, the Chinese central government has revamped the assessment index system for officials of the local government and set pollution as an important indicator; meanwhile, many environmental protection supervision actions also have been carried out in these years. Relevant empirical studies also show that the Action Plan has reduced air pollution by strengthening environmental regulation and improving regional air quality [23]. Moreover, this environmental regulation policy has also increased the size of environmental protection investment in all provinces [26].

### 2.2. Theoretical Analysis

#### 2.2.1. The Forcing Effect

To raise the pollution costs of firms and internalize the negative external effects, supervision department can strengthen regulation by levying sewage charges, environmental taxes, and fines. In this way, environmental regulations can force enterprises to increase environmental investment to reduce the costs caused by environmental pollution [27,28]. The Action Plan states that it is necessary to strengthen the comprehensive governance of air pollution in industrial enterprises and speed the elimination of under-developed production capacity, which means that enterprises with high pollution and energy consumption will face fines and even be shut down by the government. To achieve sustainable development, enterprises have to increase investment in environmental protection for reducing pollution and promoting green innovation. Although studies have pointed out that in order to reduce the regulatory burden of firms and attract more investment, the local government will not always fully fulfill environmental policies [29], the environmental protection supervision action launched by the Chinese central government and civil environmental protection supervision through the internet has reduced the possibility of collusion [30].

By formulating new market standards and adjusting market structures, environmental regulation can force enterprises to conduct green innovations, such as environmental protection technology and optimized industrial structure [31]. For example, the Action Plan states that in these big cities, such as Beijing and Shanghai, the proportion of clean fuel vehicles in new or replaced public transport vehicles every year should reach more than 60%. Liu et al. [32] revealed that China’s green industrial policy has significantly promoted the green transformation of Chinese textile industries. Such policies will accelerate the development of a new energy, the circular economy. In addition, environmental regulation will change the expectations and demands of external stakeholders, and external pressure will force managers to reconsider the possible consequences of environmental pollution [33]. Especially for listed companies, environmental regulation will change investors’ expectations, and the market valuation of high pollution and high energy consumption enterprises will be affected. Therefore, enterprises fined by the government for environmental pollution will face more severe financing constraints, which will also force investors and managers to increase investment in environmental protection and green innovation to realize the sustainable development of enterprises [21,34]. 

Although environmental regulation will undoubtedly increase the regulatory burden and cost of firms in the short term [35], government penalties and market expectations will force firms to improve green investment and control pollution emissions. Many empirical studies support this view. For example, using China’s city-level data, Zhang et al. [36] revealed that strengthening environmental legislation is an important driving force for improving firms’ investment in pollution emissions reduction and green innovation. In sum, this paper assumes that environmental regulation can stimulate firms to increase investment in pollution emissions reduction and promote green innovation (Hypothesis 1).

#### 2.2.2. The Resource Compensation Effect

Green innovation has obvious positive externalities and has characteristics of high investment and high risk. It is difficult to achieve sustainable input and development only driven by corporate social responsibility. Therefore, the Porter hypothesis indicates that environmental rules and policies can correct the negative externalities of environmental pollution, as well as account for the positive externalities of enterprise green innovation including the resource compensation effect of environmental regulation on green innovation [6]. Environmental regulation can internalize positive external effects of innovation by increasing government environmental subsidies, implementing carbon emissions trading, and other economic means. It can not only enable enterprises to avoid the cost of environmental policy compliance through green innovation but also obtain additional economic benefits to compensate for the resources consumed by enterprises for green innovation [37]. Green innovation requires a long-term investment of resources, and the lack of resources and incentives is a primary problem challenging enterprise green innovation [38]. The economic benefits brought by government subsidies are conducive to reducing managers’ concerns about the uncertainty of innovation activities and enhancing the ability to take risks [39]. Zhang and Zhao [29] found that environmental regulation can provide more financial support for enterprise research on green technology by increasing government subsidies. Liu et al. [40] found that although enterprises in eastern China face stricter environmental regulations, they can obtain more environmental subsidies and thus show a higher level of green innovation.

The resource compensation effect of environmental regulation is also reflected in that the enterprises implementing green innovation can avoid the cost of environmental regulation and obtain more competitive advantages in the market [10]. Facing increasingly stringent environmental regulations, enterprises need to pay the government for their environmental pollution behaviors to obtain production licenses and market licenses, which also constitutes the cost of compliance with the environmental regulation policies. However, enterprises that take the lead in implementing green innovation can avoid these costs by developing green production technologies and reducing environmental pollution, which can also make up for the resources consumed in green innovation [41,42]. In addition, environmental regulation drives the development of new technologies related to environmental protection and forms new market demand. It can encourage enterprises to implement green innovation by internalizing the positive externalities of green innovation with market mechanisms [43]. Moreover, for listed companies, green innovation can also boost the confidence and expectations of investors, improve the stock price, attract more investment, alleviate corporate financing constraints, and form a virtuous circle of corporate green innovation [32]. The earlier enterprises implement green innovation, the more advantages that may be obtainable.

Generally speaking, environmental regulation, through the joint action of government environmental subsidies, carbon emission trading, and other market mechanisms, can make up for the resources consumed by enterprises in the implementation of green innovation activities. Therefore, this paper assumes that environmental regulation can promote enterprise green innovation by compensating for the resources and costs needed for green innovation (Hypothesis 2).

## 3. Research Design

### 3.1. Econometric Model

The difference-in-difference (DID) model is used to evaluate the policy effects in empirical research. It compares the differences between the pilot area (experimental group) and the non-pilot area (control group) before and after the policy implementation and eliminates differences caused by trends over time so that the pure policy effect can be evaluated.

The Action Plan states that the comprehensive rectification of volatile organic compounds will be implemented in the petrochemical, organic chemical, surface coating, packaging, and printing industries. This article defines these industries as key regulatory industries and defines other industries as non-key regulatory industries. On the basis of the DID model, this article sets the following econometric model to evaluate the effects of the Action Plan on corporate green innovation.

(1) $${\mathrm{GreenPatent}}_{it}=\beta_{0}+\beta_{1}\times {\mathrm{Post}}_{t}\times {\mathrm{Pollution}}_{i}+X_{it}+\mu_{i}+\gamma_{t}+\varepsilon_{it}$$

where i is the company’s industry; t is the time; $X_{it}$ is a vector of the control variables; $\varepsilon_{it}$ is an error term;$\;\mu_{i}$ and $\gamma_{t}$ are the fixed effects for industry and year, respectively; $\beta_{0}$ is the intercept; and $\beta_{1}$ is the policy effect to be estimated. This article compares the impact of the policy in industries with different intensities of regulation, as well as the heterogeneity of the policy’s impact on different types of corporate green patents.

### 3.2. Variables

The explained variable ${\mathrm{GreenPatent}}_{it}$ is measured by the amounts of green patent applications of the company [32]. For green innovation (GreenPatent), we identified and collected the amounts of green patent applications of enterprises to standardize green innovation according to the standard of WIPO. Compared with the amounts of total patents issued by listed companies, adopting green patents is more effective in eliminating the influence of other non-observable factors [44]. Generally, patents are divided into invention patents, utility patents, and design patents, and green patents usually involve only invention patents and utility patents. Therefore, in this paper, we used the sum of green invention patents and green utility patents of listed companies to measure a company’s green innovation level. In the heterogeneity analysis, this paper further explored the impact of environmental regulation on the above two types of green patents. 

The interactive term of ${\mathrm{Post}}_{t}\times {\mathrm{Pollution}}_{i}$ is the core independent variable that is constructed by two dummy variables, namely, ${\mathrm{Post}}_{t}$ and ${\mathrm{Pollution}}_{i}$. Because the Action Plan was released in September 2013, we therefore define ${\mathrm{Post}}_{t}\;$ as 1 in the year 2014 and later years and the years before 2014 as equal to 0. ${\mathrm{Pollution}}_{i}$ is an indicator of industry pollution attributes. We define the heavily polluting industries as equal to 1 and non-heavy polluting industries as equal to 0. 

The focus of the research is the coefficient of ${\;\mathrm{Post}}_{t}\times {\mathrm{Pollution}}_{i}$. Generally, the classic DID model examines the difference between regulated industries and non-pilot industries before and after the policy is implemented. That is, the difference in the promotion of green patents of enterprises in polluting and non-polluting industries is due to the Action Plan.

Referring to previous literature [32,45], we add the following control variables to cover firm characteristics: (1) firm size (Size), defined as the log form of total assets; (2) capital structure (LEV), defined as the ratio of total liabilities to total assets; (3) state-owned enterprises (STATE), which is equal to one for state-owned enterprises and zero for non-state-owned enterprises; (4) amounts of employees (NUM), defined as the natural logarithm of the number of employees at the end of the year; (5) firm age (Age), represented by the year of investigation minus year of establishment, which is equal to 2017 minus year of establishment; (6) corporate financing constraints (SA), with SA = 0.043 × Size^2^ − 0.737 × Size − 0.040 × A (According to previous literature, the measurement methods of corporate financing constraints usually include the SA, KZ, and WW indexes; this paper used the SA index to measure corporate financing constraints [46]. In the formula, Size is the enterprise scale expressed by the natural logarithm of the total assets of the enterprise standardized in millions, and Age is the age of the enterprise.).

The definitions of related variables are presented in Table 1.

### 3.3. Samples and Data

To investigate the effect of the Action Plan on green innovation behaviors of firms, we constructed the dataset using China’s listed companies in A-Share. The dataset begins from 2009 and ends in 2017, which is mainly because (1) the data of all variables are hard to collect from the annual reports before 2009, and (2) in order to carry out an empirical simulation with the DID model, the beginning year of the dataset should be more than 2 years earlier than the year of policy implementation, and the ending year should be more than 2 years after the policy implementation. For the data source, the data on green patents of listed companies were collected and compiled from SIPO of China; the corporate environmental subsidy data were collected from annual financial reports, and all other variables are obtained from the China Stock Market and Accounting Research Database (CSMAR) and the WIND database. The classification of polluting industries is based on the key industries involved in the Action Plan as the standard. 

The samples were screened according to the following criteria: First, because the financial treatment of financial industry companies, GEM listed companies, and small- and medium-sized listed companies are significantly different from other companies, the above companies were eliminated. Second, ST companies were eliminated. Third, we removed samples with missing data. Fourth, we removed abnormal values of financial indicators. In addition, this paper also conducted a 1% tail reduction on the sample size according to the value of the dependent variable. Because the social responsibility report of listed companies does not have to be disclosed, 11,241 sample observations were obtained. Data processing and analysis were completed in Stata15.

Table 2 presents the descriptive statistics of all variables. The mean of corporate green patents was 0.203. Obviously, during the sample period, the amount of green patent applications in China was not large, which is consistent with the situation of China and was mainly because of the lack of policies to stimulate green innovation. The average number of green invention patents applied for by enterprises was 0.097, with the average number of green utility model patents at 0.157. This is consistent with the current situation in Chinese current patent law that the requirements for applying for utility model patents are lower than for applying for invention patents. The differences in the company’s total assets (*Size*), number of employees (*NUM*), and financing constraints (*LEV* and *SA*) are more obvious, laying the foundation for the subsequent research.

## 4. Empirical Results

### 4.1. Baseline Regression Results

This part involved the conducting of the regression analysis using model (1). Column 1 of Table 3 only regresses the interactive term Pollution × Post; column 2 adds all control variables to the model; column 3 controls the fixed effects of year and industry; and column 4 adds industry-level clustering to column 3. It is clear that the interactive term had a significant positive effect on the Green Patent in all models, which means that the Action Plan can stimulate firms’ green innovation behaviors effectively. This result indicates that the previous hypothesis in this article is supported—the Action Plan increased the number of green patents in the pilot industries. It also provides new evidence for the Porter hypothesis. 

In addition, for the control variables, the number of employees (NUM) and financing constraints (SA) of an enterprise promote green innovation. Usually, implementing R&D activities requires many human and financial resources; hence, a company with more employees and finances will have a greater ability and incentive to carry out R&D activities. Moreover, it can be seen that the effect of LEV was not significant. This is because an enterprise can finance loans or use its funds for R&D innovation and there are various financial subsidies. Therefore, the company LEV had no direct relationship with the company’s green innovation.

### 4.2. Robustness Checks

#### 4.2.1. Dynamic DID

When adopting the DID model, it is necessary to test the parallel trend between the samples in the control and treatment groups. The parallel trend test is also a necessary step in identifying whether the effect is caused by the policy. Here, we used the method of event analysis to test the parallel trend. As shown in Table 4, we used the dummy of pre_1 and pre_2 to represent the first year, the first two years and earlier years before the Action Plan respectively, and used the dummy of post_1 and post_2 to represent the first year, the second year and later years after the Action Plan respectively. Meanwhile, we used the dummy of current to represent the year that the Action Plan was carried out, namely, 2014. 

The results of the inspection are shown in Table 4. In the two years before the implementation of the Action Plan, the level of firms’ green innovation in regulated regions and industries did not increase significantly until 2014, the policy implementation year, and the following two years. This supports the assumption of parallel trends when the DID model is applied. 

#### 4.2.2. Bootstrap

The bootstrap method is a computer simulation that is commonly used to analyze statistics that require large samples—a new sample is randomly selected from the original dataset with a replacement. We used the method of random sampling to repeatedly extract 500 times and calculate their mean to construct a new sample, which is conducive to constructing a more steady and robust dataset. Therefore, we used a bootstrap sampling method for the robustness test. As shown in Table 5, this policy had a significant positive effect on the amount of a firm’s total green patent application and green utility patent application but had no significant effect on green invention patent application. These results are consistent with the previous results.

#### 4.2.3. Propensity Score Matching Estimation

To further address the endogenous issue, we used the Propensity Score Matching (PSM) method to construct a comparable control group (Table 6). The idea of PSM is based on similarity, calculated from various observable characteristics. In this, total assets, number of employees, financing constraints, and the asset–liability ratio of enterprises were used as matching indexes to conduct caliper matching for enterprises in regulated and non-regulated industries by year. The regression results are consistent with other findings in this paper.

#### 4.2.4. Placebo Test

To corroborate that the inference was not due to random chance, we conducted a placebo test by creating a placebo treatment (Table 7). In this, the treatment and control groups were randomly selected from the sample at a ratio of 3:7 to investigate whether potential omitted variables will affect the promotion effect. The regression results showed that the estimated coefficient of the cross-product term was not significant, which supports the previous conclusions.

## 5. Further Analysis

### 5.1. Analysis of Heterogeneity at the Regional Level of Key Regulations

Here, we constructed a DDD (DDD stands for triple difference method, and detailed description of this method can be seen in the research made by Olden and Moen [47]) model to explore the impact of the Action Plan on enterprises’ green patents in regions with different levels of regulation. *Region* represents the dummy variable of the area where the Action Plan is implemented. In this paper, the regions (specifically, the Beijing–Tianjin–Hebei, Yangtze River Delta, and Pearl River Delta) that implemented the policy for fine particulate matter ($PM_{2.5}$) were set as pilot regions, and the value was 1. Other cities were set as non-pilot areas, and the value was 0. The interaction term was inserted into the model regression, and the regression results are shown in Table 8.

In this part of the research, the focus was on the coefficients of the triple interaction terms. The results in Table 8 show that after adding the regional variable *Region*, the coefficient of the triple interaction term increased compared with the coefficient of the previous double interaction term *Pollution* × *Post* and was significantly positive at the 1% level. If the area of a company is a key regulatory area of the Action Plan, it will have a positive impact on the number of applications for green patents. This is because the governance requirements of key regulatory areas are more stringent, and companies will have stronger motivation to carry out innovation and improve production technology and equipment.

### 5.2. Analysis of Heterogeneity of Green Patent Types

For Chinese current patent law, the requirements for applying for utility model patents are lower than those for applying for invention patents. According to the existing literature, some companies that are motivated by industrial policies have selectively increased the number of patent applications to obtain more financial subsidies or tax incentives, especially with the significant increase in the number of non-invention patents [48]. Such an industrial policy causes enterprises to pursue “quantity” while ignoring “quality” and to innovate for “seeking support”. This ultimately cannot effectively improve competitiveness.

We divided green patents into green invention patents and green utility model patents to further examine the heterogeneity of the Action Plan to corporate green innovation. We collected the green invention patents and utility model patent application data from the SIPO database. Invention patents (*Invention*) were defined as the natural logarithm of the number of green invention patents plus 1. A utility model patent (*Utility*) was defined as the natural logarithm of the number of green utility model patents plus 1. We substituted them into model (1) for regression analysis. From the results in columns (1) and (2) of Table 9, it can be seen that the Action Plan had a significant promotion effect on the number of green utility model patents of enterprises but no significant effect on the number of green invention patent applications. This result is similar to previous literature reports. When companies innovate, the first choice is the least difficult innovation project. This may also be related to the situation that enterprises in the pilot industries in the Action Plan have historically had a large lack of green innovation and technology.

### 5.3. Analysis of the Heterogeneity of the Ownership

In China, the special connection between SOEs (state-owned enterprises) and the government usually makes it easier for SOEs to obtain fiscal subsidies from the government and escape various regulations [49]. Hence, environmental regulation is likely to generate different incentives for green innovation behaviors between SOES and private firms because their costs and benefits obtained from environmental regulation are likely to be different. To discriminate the different effects between these two types of firms, we divided the firm samples into SOEs and private firms for group regression.

As shown in Table 10, the interaction item had no significant impact on both types of firms when we used green invention patents as the dependent variable. However, it had a significant positive effect on green innovation of SOES when we used green utility patents as the dependent variable, but its effect on private firms was still not significant. Because R&D activities are time consuming, expensive, and risky, the ability of R&D financial support for SOEs was relatively stronger than that of non-SOEs. Therefore, SOEs are more capable of investing resources in green patent research and development. On the other hand, the Action Plan policy focuses on eliminating high-energy-consuming and high-polluting enterprises, increasing the use of clean energy, implementing energy-saving emission reduction mechanisms, and using laws and regulations to “force” enterprises to transform and upgrade. Therefore, regardless of the ownership of an enterprise, if it wants to survive such strict environmental regulation, it must carry out substantive innovation, rather than selectively carry out strategic innovation activities (such as utility model patents).

## 6. Mechanism Analysis

In the former section, we revealed that environmental regulation can stimulate firms’ green innovation successfully. However, the mechanisms behind this effect are still worth further study. Here, we used the environmental protection subsidies obtained by enterprises to test the resource compensation effect and firm’s investment in pollution control and governance to test the forcing effect. Environmental protection subsidies refer to fiscal subsidies given by the government for firms’ environmental protection activities. We collected the data from the detailed collation of non-operating income in companies’ annual financial reports. There are two types of government environmental protection subsidies. One is a bonus, such as awards for reduction of energy consumption and pollution emissions, and the other is environmental-protection-related honors from the government to enterprises. This type of financial subsidy is usually small. The other is non-bonus forms of environmental protection subsidies, such as interest discounts or financial support to support energy saving and environmental protection research. The environmental protection subsidy (***EnvSub***) is measured by the natural logarithm of the total environmental protection subsidies.

According to the measurement method of Liu and Zhang [50], the total asset scale is taken as the natural logarithm to measure the overall capital investment of the enterprise, whereas the fixed asset is taken as the logarithm to measure the corporate productive capital investment. Corporate environmental protection investment (***Invest***) is measured by the natural logarithm of the difference between the two. Considering that environmental regulation policies may have a time lag, we also used the t + 1 period of environmental protection investment (***FutureInvest***) to test whether environmental regulations will promote corporate green innovation by influencing corporate environmental investment in future years. The specific regression analysis results are shown in Table 11.

The empirical results show that “Pollution × Post × EnvSub” had a significant positive effect on the amount of green patent application. The result indicates that the effect of the Action Plan on corporate green innovation was pronounced, which also means the Porter hypothesis holds for this dataset. Reasonable environmental regulations can promote corporate innovation activities. Environmental resources are public products, and it is difficult to achieve balance only by the regulation of market mechanisms. Therefore, to control environmental pollution, government environmental and economic policies are necessary. From the results in columns 2 and 3 of Table 11, it can be seen that whether it is an environmental protection investment in an enterprise in period t or environmental capital investment in period t + 1, both of the interactive terms had a significant positive effect on the amount of firms’ green patent application. These results indicate that environmental regulations can stimulate and force firms to improve investment in green development, thus promoting green innovation. As the Action Plan is intended to reduce pollutant emissions in key governance areas and industries, this shows that companies are increasing their investment in cleaner production, energy saving, and environmental protection assets.

## 7. Conclusions and Policy Implications

Through theoretical analysis, this paper assumes that environmental regulation can promote green innovation through the forcing effect and resource compensation effect. In order to test this hypothesis, this paper regarded China’s Air Pollution Prevention and Control Action Plan released in 2014 as a quasi-natural experiment to strengthen the intensity of environmental control and produced an empirical analysis using the data of Chinese A-Share listed companies. The empirical results revealed the following conclusions: (1) The green innovation level of A-Share listed companies in China has been improved significantly through strengthening environmental regulation as represented by this Action Plan. (2) Compared with non-regulated industries, the amount of green patent applications has been improved by 5.4% in firms of regulated industries. Meanwhile, the promotion effect is more obvious in key areas that are more strictly controlled by this policy. (3) Compared with private owned firms, the Action Plan has a stronger promotion effect on the green innovation of state-owned enterprises. (4) The forcing effect and resource compensation effect are mechanisms of environment regulation that promote enterprise green innovation. China’s environmental regulation policy can have a resource compensation effect by increasing environmental protection subsidies for enterprises, and it can force enterprises to increase environmental protection investment and promote enterprise green innovation through the forcing effect.

It is undeniable that this article still has some shortcomings. For example, the relationship between these two issues is complex, and there may be multiple affecting mechanisms. However, this paper only analyzed its forcing effect and resource compensation effect from the perspective of environmental investment and environmental subsidies obtained by enterprises. Other influence channels can be included, such as new product development, digital upgrading, shadow economic activities, and the energy use structure of enterprises. In addition, the Action Plan contains command type and market-driven environmental regulation policies, but it is difficult to distinguish the effect of these two types of policies on green innovation.

This paper has policy implications for countries that intend to promote enterprise green innovation by carrying out environmental regulations. First, strengthening environmental regulation can stimulate firms to pay more attention to green innovation and improve their ability to realize green development. Thus, the government should actively introduce and implement environmental protection policies and strengthen environmental regulation. Second, the government should recognize and utilize the forcing effect and resource compensation effect of environmental regulation. While using the command environmental regulation policy to force enterprises to reduce environmental pollution and strengthen environmental investment, the functions of market-driven environmental regulation should not be ignored. It can be used to increase the financial subsidies and economic benefits that enterprises can obtain through green innovation and provide the endogenous impetus for green innovation. Third, the fairness of environmental policy is an issue that deserves attention. SOEs have greater advantages in escaping environmental regulation and obtaining subsidies, which will lead to market unfairness. Thus, it is necessary to keep the consistency of enforcement and compensation of environmental regulation in state-owned enterprises and private enterprises consistent and to create a favorable system for improving the green innovation level of private enterprises. Finally, it is important to discriminate the heterogeneity of different industries and regions when implementing environmental protection policies. For heavily polluted industries and regions, the government should implement stricter environmental regulation policies to force these enterprises to increase environmental investment and strengthen green innovation.

## Figures and Tables

**Table 1:** Definitions of variables.

Variable	Symbol	Measurement of Variables
The amounts of green patent applications of enterprises	*GreenPatent*	ln(the amounts of green invention patents + the number of green invention patents1)
Green invention patents	*Invention*	ln(the number of green invention patents + 1)
Green utility model patents	*Utility*	ln(the number of green utility model patents + 1)
Indicators of industry pollution attributes	*Pollution*	1 indicates a polluting industry and 0 otherwise
Capital structure	*LEV*	The ratio of total liabilities to total assets
Number of employees	*NUM*	The natural logarithm of the actual number of employees at the end of the year
Firm age	*AGE*	The year minus the year the company was established
Total assets	*SIZE*	The log form of total assets
Property rights	*STATE*	1 for state-owned and 0 otherwise
Corporate financing constraints	*SA*	SA = 0.043 × Size^2^ − 0.737 × Size − 0.040 × Age

**Table 2:** Summary statistics of all variables.

	(1)	(2)	(3)	(4)	(5)
	Obs	Mean	Std. Dev.	Min	Max
Size	11,241	22.418	1.395	19.376	26.426
Num	11,241	7.826	1.461	3.738	11.412
GreenPatent	11,239	0.203	0.593	0	3.296
Invention	11,239	0.097	0.377	0	2.398
Utility	11,239	0.157	0.501	0	2.833
Pollution	11,241	0.147	0.354	0	1
SA	8519	21.605	2.773	15.792	30.347
Region	11,241	0.480	0.500	0	1
Age	8519	21.276	4.354	9	33
LEV	11,241	0.502	0.206	0.019	1.636

**Table 3:** Environmental regulation and corporate green innovation.

Variable	(1)	(2)	(3)	(4)
	GreenPatent	GreenPatent	GreenPatent	GreenPatent
Post × Pollution	0.087 ***	0.066 ***	0.054 ***	0.054 *
	(0.015)	(0.016)	(0.018)	(0.032)
Size		−0.760 ***	−0.734 ***	−0.734 ***
		(0.096)	(0.152)	(0.252)
NUM		0.032 ***	0.018 *	0.018 *
		(0.008)	(0.010)	(0.010)
SA		0.417 ***	0.390 ***	0.390 ***
		(0.049)	(0.078)	(0.132)
LEV		−0.039	−0.035	−0.035
		(0.033)	(0.038)	(0.044)
Constant	0.195 ***	7.969 ***	8.031 ***	8.031 ***
	(0.013)	(1.100)	(1.737)	(2.810)
Year	N	N	Y	Y
Industry	N	N	Y	Y
Firm Clustered	N	N	N	Y
Observations	11239	8517	8517	8517
R-squared			0.035	0.035
Number of ID	1661	1318	1318	1318

Notes: Standard errors clustered at the firm level are reported in parentheses. *** and * indicate significance at the 1% and 10% levels, respectively.

**Table 4:** Environmental regulation and corporate green innovation: dynamic DID.

Variable	(1)	(2)	(3)
	GreenPatent	GreenPatent	GreenPatent
pre_2 × pollution	0.033	0.035	0.017
	(0.024)	(0.025)	(0.029)
pre_1 × pollution	0.038	0.034	0.007
	(0.024)	(0.025)	(0.029)
current × pollution	0.107 ***	0.094 ***	0.068 **
	(0.023)	(0.024)	(0.028)
post_1 × pollution	0.117 ***	0.097 ***	0.071 **
	(0.023)	(0.024)	(0.028)
post_2 × pollution	0.068 ***	0.043 *	0.038
	(0.023)	(0.025)	(0.029)
Size		−0.760 ***	−0.731 ***
		(0.096)	(0.152)
Num		0.032 ***	0.018 *
		(0.008)	(0.009)
SA		0.417 ***	0.388 ***
		(0.049)	(0.078)
LEV		−0.041	−0.034
		(0.033)	(0.038)
Constant	0.193 ***	7.980 ***	7.994 ***
	(0.013)	(1.100)	(1.738)
Year	N	N	Y
Industry	N	N	Y
Observations	11,239	8517	8517
R-squared			0.035
Number of ID	1661	1318	1318

Notes: Standard errors are reported in parentheses. ***, ** and * indicate significance at the 1%, 5%, and 10% levels, respectively.

**Table 5:** Environmental regulation and corporate green innovation: bootstrap.

Variable	(1)	(2)	(3)
	GreenPatent	Invention	Utility
Pollution × Post	0.054 **	0.017	0.064 *
	(0.025)	(0.031)	(0.037)
Size	−0.734 ***	−0.538 *	−0.507 *
	(0.115)	(0.312)	(0.267)
NUM	0.018	0.009 *	0.016 *
	(0.018)	(0.005)	(0.009)
SA	0.390 ***	0.291 *	0.267 *
	(0.063)	(0.163)	(0.140)
LEV	−0.035	−0.026	−0.036 **
	(0.035)	(0.025)	(0.017)
Constant	8.031 ***	5.848 *	5.508 *
	(1.591)	(3.513)	(3.052)
Year	Y	Y	Y
Industry	Y	Y	Y
Observations	8517	8517	8517
R-squared	0.035	0.026	0.030
Number of ID	1318	1318	1318

Notes: Standard errors are reported in parentheses. ***, ** and * indicate significance at the 1%, 5%, and 10% levels, respectively.

**Table 6:** Environmental regulation and corporate green innovation: PSM estimation.

Variable	(1)	(2)
	GreenPatent	GreenPatent
Pollution × Post	0.0438 **	0.0535 **
	(2.69)	(3.05)
Size		−0.759 ***
		(−5.13)
Num		0.0180 *
		(2.03)
SA		0.400 ***
		(5.27)
LEV		−0.0349
		(−0.94)
Constant		8.400 ***
		(5.00)
Year	Y	Y
Industry	Y	Y
Observations	11,239	8517
R-squared	0.035	0.026
Number of ID	1318	1318

Notes: Standard errors are reported in parentheses. ***, ** and * indicate significance at the 1%, 5%, and 10% levels, respectively.

**Table 7:** Environmental regulation and corporate green innovation: placebo test.

Variable	(1)	(2)
	GreenPatent	GreenPatent
Pollution × Post	−0.002	0.017
	(−0.16)	(1.09)
Size		−1.221 ***
		(−7.19)
Num		0.021 **
		(2.03)
SA		0.638 ***
		(7.33)
LEV		−0.034
		(−0.80)
Constant		8.400 ***
		(5.00)
Year	Y	Y
Industry	Y	Y
Observations	11,215	8496
R-squared	0.0309	0.0413
Number of ID	1318	1318

Notes: Standard errors are reported in parentheses. ***, ** and * indicate significance at the 1%, 5%, and 10% levels, respectively.

**Table 8:** Analysis of the heterogeneity of key regulatory regions.

Variable	(1)	(2)	(3)	(4)
	GreenPatent	GreenPatent	GreenPatent	GreenPatent
Post × Region × Pollution	0.135 ***	0.115 ***	0.108 ***	0.108 **
	(0.021)	(0.022)	(0.024)	(0.051)
Size		−0.743 ***	−0.693 ***	−0.693 ***
		(0.096)	(0.152)	(0.249)
NUM		0.0321 ***	0.017 *	0.0174 *
		(0.008)	(0.009)	(0.010)
SA		0.409 ***	0.369 ***	0.369 ***
		(0.049)	(0.078)	(0.131)
LEV		−0.038	−0.032	−0.032
		(0.033)	(0.038)	(0.044)
Constant	0.196 ***	7.785 ***	7.577 ***	7.577 ***
	(0.013)	(1.100)	(1.739)	(2.777)
Year	N	N	Y	Y
Industry	N	N	Y	Y
Firm Clustered	N	N	N	Y
Observations	11,239	8517	8517	8517
R-squared			0.037	0.037
Number of ID	1661	1318	1318	1318

Notes: Standard errors are reported in parentheses. ***, ** and * indicate significance at the 1%, 5%, and 10% levels, respectively.

**Table 9:** Analysis of heterogeneity of green patent types.

Variable	(1)	(2)	(3)	(4)
	Invention	Invention	Utility	Utility
Pollution × Post	0.016	0.017	0.072 ***	0.064 **
	(0.012)	(0.023)	(0.014)	(0.027)
Size	−0.428 ***	−0.538 ***	−0.600 ***	−0.507 **
	(0.064)	(0.203)	(0.082)	(0.214)
Num	0.022 ***	0.009	0.025 ***	0.016 *
	(0.005)	(0.006)	(0.007)	(0.008)
SA	0.237 ***	0.291 ***	0.329 ***	0.267 **
	(0.033)	(0.106)	(0.041)	(0.112)
LEV	−0.013	−0.026	−0.0410	−0.036
	(0.024)	(0.033)	(0.029)	(0.037)
Constant	4.400 ***	5.848 **	6.305 ***	5.508 **
	(0.739)	(2.271)	(0.937)	(2.386)
Year	Y	Y	Y	Y
Industry	Y	Y	Y	Y
Firm Clustered	N	Y	N	Y
Observations	8517	8517	8517	8517
R-squared		0.026		0.030
Number of ID	1318	1318	1318	1318

Notes: Standard errors are reported in parentheses. ***, ** and * indicate significance at the 1%, 5%, and 10% levels, respectively.

**Table 10:** Analysis of the heterogeneity of the ownership.

Variable	(1)	(2)	(3)	(4)
	Invention		Utility	
	SOE	Non-SOE	SOE	Non-SOE
Pollution × Post	0.006	0.014	0.073 ***	0.0394
	(0.017)	(0.025)	(0.020)	(0.029)
Size	−0.326 ***	−0.348 ***	−0.362 ***	−0.585 ***
	(0.092)	(0.090)	(0.114)	(0.114)
Num	0.008	0.008	0.017 *	0.013
	(0.008)	(0.008)	(0.010)	(0.010)
SA	0.195 ***	0.205 ***	0.212 ***	0.327 ***
	(0.046)	(0.047)	(0.057)	(0.059)
LEV	−0.020	−0.068 **	−0.058	−0.045
	(0.036)	(0.034)	(0.043)	(0.041)
Constant	3.078 ***	3.390 ***	3.392 **	5.922 ***
	(1.076)	(1.026)	(1.333)	(1.297)
Year	Y	Y	Y	Y
Industry	Y	Y	Y	Y
Observations	5411	3106	5411	3106
Number of ID	790	617	790	617

Notes: Standard errors are reported in parentheses. ***, ** and * indicate significance at the 1%, 5%, and 10% levels, respectively.

**Table 11:** Environmental regulation and corporate green innovation: mechanisms analysis.

Variable	(1)	(2)	(3)
	GreenPatent	GreenPatent	GreenPatent
Size	−0.941 ***	−0.666 ***	−0.687 ***
	(0.257)	(0.093)	(0.095)
Num	−0.000	0.0242 ***	0.0236 ***
	(0.027)	(0.008)	(0.008)
SA	0.523 ***	0.372 ***	0.384 ***
	(0.128)	(0.047)	(0.048)
LEV	−0.173 *	−0.038	−0.039
	(0.093)	(0.034)	(0.037)
Pollution × Post × EnvSub	0.008 **		
	(0.003)		
Pollution × Post × Invest		0.003 ***	
		(0.001)	
Pollution × Post × FutureInvest			0.003 ***
			(0.001)
Constant	10.04 ***	6.740 ***	6.944 ***
	(2.973)	(1.068)	(1.099)
Year	Y	Y	Y
Industry	Y	Y	Y
Observations	1485	8517	8308
Number of ID	332	1318	1314

Notes: Standard errors are reported in parentheses. ***, ** and * indicate significance at the 1%, 5%, and 10% levels, respectively.

## Data Availability

Data available on request.

## References

[1] Cheng Z., Kong S. (2022). The effect of environmental regulation on green total-factor productivity in china’s industry. Environ. Impact Assess. Rev..

[2] Yale University of Environmental Law & Policy 2018 Environmental Performance Index. https://envirocenter.yale.edu/2018-environmental-performance-index.

[3] Zhang M., Liu X., Sun X., Wang W. (2020). The influence of multiple environmental regulations on haze pollution: Evidence from China. Atmos. Pollut. Res..

[4] Peng H., Shen N., Ying H., Wang Q. (2021). Can environmental regulation directly promote green innovation behavior?—based on situation of industrial agglomeration. J. Clean. Prod..

[5] Liu B., Wang X.Z. (2021). The Risk Compensation Effect of Enterprise Green Innovation on Stock Returns. Econ. Manag..

[6] Porter M., Van-der Linde C. (1995). Toward a New Conception of the Environment-Competitiveness Relationship. J. Econ. Perspect..

[7] Du K., Cheng Y., Yao X. (2021). Environmental regulation, green technology innovation, and industrial structure upgrading: The road to the green transformation of Chinese cities. Energy Econ..

[8] Reiner A.D. (2020). European industrial energy intensity: Innovation, environmental regulation, and price effects. Energy J..

[9] Ravetti C., Theoduloz T., Valacchi G. (2020). Buy Coal or Kick-Start Green Innovation? Energy Policies in an Open Economy. Env. Resour. Econ..

[10] Zhong Z.Q., Peng B.H. (2022). Can environmental regulation promote green innovation in heavily polluting enterprises? Empirical evidence from a quasi-natural experiment in China. Sustain. Prod. Consum..

[11] Song P., Chen M.Y., Mao X.Q. (2022). Does Central Environmental Protection Inspection Promote Green Innovation of Heavily Pollution Companies?—Evidence from Green Patent Data of Chinese Listed Companies. China Environ. Manag..

[12] Shleifer A., Vishny R.W. (1994). Politicians and Firms. Q. J. Econ..

[13] Hottenrott H., Rexhäuser S., Veugelers R. (2016). Organisational change and the productivity effects of green technology adoption. Resour. Energy Econ..

[14] Yu W., Ramanathan R., Nath P. (2016). Environmental pressures and performance: An analysis of the roles of environmental innovation strategy and marketing capability. Technol. Forecast. Soc. Chang..

[15] Kneller R., Manderson E. (2012). Environmental regulations and innovation activity in UK manufacturing industries. Resour. Energy Econ..

[16] Barbieri N. (2015). Investigating the impacts of technological position and European environmental regulation on green automotive patent activity. Ecol. Econ..

[17] Fan F., Lian H., Liu X., Wang X. (2020). Can environmental regulation promote urban green innovation efficiency? an empirical study based on Chinese cities. J. Clean. Prod..

[18] Song M., Xie Q., Wang S., Zhang H. (2020). Could environmental regulation and R&D tax incentives affect green product innovation?. J. Clean. Prod..

[19] Zhao X., Sun B.W. (2016). The influence of Chinese environmental regulation on corporation innovation and competitiveness. J. Clean. Prod..

[20] Chen X.H., Yi N., Zhang L., Li D.Y. (2018). Does institutional pressure foster corporate green innovation? Evidence from China’s top 100 companies. J. Clean. Prod..

[21] Bu M.L., Qiao Z.Z., Liu B.B. (2020). Voluntary environmental regulation and firm innovation in China. Econ. Model..

[22] Fan H., Zhao C., Yang Y. (2020). A comprehensive analysis of the spatio-temporal variation of urban air pollution in China during 2014–2018. Atmos. Environ..

[23] Wu Z.N., Yin Y.K. (2022). Does the air pollution prevention and control action play a good role in the “blue sky defense” of resource-based cities?. Rev. Ind. Econ..

[24] Yang S.Y., Wang F., Liu N. (2020). Evaluation of the implementation effect of the Air Pollution Prevention and Control Action Plan: Based on the DID Method. Chin. J. Popul. Resour. Environ..

[25] Suo L.M., Kan Y.Q. (2021). Understanding the Diversity of China’s Air Pollution Collaborative Governance Organizations: Type Differences and Selection Logic. J. South China Norm. Univ..

[26] Zhou D., Peng X.L., Huang Q. (2021). Can imperative environmental regulations promote R&D and innovation activities of enterprises—Take “Ten Atmospheres” as an example. Sci. Res. Manag..

[27] Xie R.H., Yuan Y.J., Huang J.J. (2017). Different Types of Environmental Regulations and Heterogeneous Influence on “Green” Productivity: Evidence from China. Ecol. Econ..

[28] Acemoglu D.P., Aghion L., Hemous D. (2012). The Environment and Directed Technical Change. Am. Econ. Rev..

[29] Chakrabortya P., Chatterjee C.B. (2017). Does environmental regulation indirectly induce upstream innovation? New evidence from India. Res. Policy.

[30] Zhang B.C., Zhao S.K. (2022). Research on the Impact of Government Subsidies on Green Innovation of Enterprises—The Moderating Effect of Political Connection and Environmental Regulation. Sci. Res. Manag..

[31] Li J., Du Y.X. (2020). Spatial effect of Environmental Regulation on Green Innovation Efficiency——Evidence from Prefectural-level Cities in China. J. Clean. Prod..

[32] Liu Y., Wang A., Wu Y. (2021). Environmental regulation and green innovation: Evidence from China’s new environmental protection law. J. Clean. Prod..

[33] Li Q.Y., Xiao Z.H. (2020). Heterogeneous Environmental Regulation Tools and Green Innovation Incentives: Evidence from Green Patents of Listed Companies. Econ. Res. J..

[34] Xu X.D., Zeng S.X., Zhou H.L., Shi J.J. (2016). The Impact of Corporate Environmental Violation On Shareholders’ Wealth: A Perspective Taken From Media Coverage. Bus. Strategy Environ..

[35] Petroni G., Bigliardi B., Galati F. (2019). Rethinking the Porter Hypothesis: The Underappreciated Importance of Value Appropriation and Pollution Intensity. Rev. Policy Res..

[36] Zhang Q., Zheng Y., Kong D.M. (2019). Regional environmental governance pressure, executive experience and enterprise environmental protection investment—A quasi natural experiment based on Ambient Air Quality Standard. Econ. Res. J..

[37] Montmartin B., Herrera M. (2015). Internal and External Effects of R&D Subsidies and Fiscal Incentives: Empirical Evidence Using Spatial Dynamic Panel Models. Res. Policy.

[38] Manso G. (2011). Motivating Innovation. J. Financ..

[39] Stiglitz J. (2015). Leaders and Followers: Perspectives on the Nordic Model and the Economics of Innovation. J. Public Econ..

[40] Liu D.Y., Chen T., Liu X.Y. (2019). Do more subsidies promote greater innovation? Evidence from the Chinese electronic manufacturing industry. Econ. Model..

[41] Lovely M., Popp D. (2011). Trade, Technology, and the Environment: Does Access to Technology Promote Environmental Regulation. J. Environ. Econ. Manag..

[42] Gao X., He Z.H., Zhang F.Y. (2022). How to Improve Region-Level Green Technology Innovation by Government Subsidies and Environmental Regulations?—A Research on Co-movement Effects from Configuration. RD Manag..

[43] Ouyang X.L., Li Q., Du K.R. (2020). How does environmental regulation promote technological innovations in the industrial sector? Evidence from Chinese provincial panel data. Energy Policy.

[44] Popp D. (2006). International Innovation and Diffusion of Air Pollution Control Technologies: The Effects of NO_X_ and SO_2_ Regulation in the US, Japan, and Germany. J. Environ. Econ. Manag..

[45] Yuan B., Xiang Q. (2018). Environmental regulation, industrial innovation and green development of Chinese manufacturing: Based on an extended CDM model. J. Clean. Prod..

[46] Hadlock C.J., Pierce J.R. (2010). New Evidence on Measuring Financial Constraints: Moving Beyond the KZ Index. Rev. Financ. Stud..

[47] Olden A., Men J. (2022). The Triple Difference Estimator. Econom. J..

[48] Li W.J., Zheng M.N. (2016). Is it Substantive Innovation or Strategic Innovation?—Impact of Macroeconomic Policies on Micro-enterprises’ Innovation. Econ. Res. J..

[49] Piotroski J., Wong T.J. (2011). Capitalizing China.

[50] Liu Y.W., Zhang Z.Z. (2016). Research on the Counterbalance Mechanism of Block holders, Chairman Tenure and Investment Extrusion. Nankai Bus. Rev..

